# Arrhythmogenic Cardiomyopathy—Current Treatment and Future Options

**DOI:** 10.3390/jcm10132750

**Published:** 2021-06-22

**Authors:** Federico Migliore, Giulia Mattesi, Alessandro Zorzi, Barbara Bauce, Ilaria Rigato, Domenico Corrado, Alberto Cipriani

**Affiliations:** Department of Cardiac, Thoracic and Vascular Sciences and Public Health, University of Padova, Via Giustiniani 2, 35128 Padova, Italy; federico.migliore@unipd.it (F.M.); g.mattesi17@gmail.com (G.M.); alessandro.zorzi@unipd.it (A.Z.); barbara.bauce@unipd.it (B.B.); ilaria.rigato@unipd.it (I.R.); alberto.cipriani@unipd.it (A.C.)

**Keywords:** arrhythmogenic cardiomyopathy, risk stratification, drug therapy, implantable cardioverter defibrillator, catheter ablation, treatment

## Abstract

Arrhythmogenic cardiomyopathy (ACM) is an inheritable heart muscle disease characterised pathologically by fibrofatty myocardial replacement and clinically by ventricular arrhythmias (VAs) and sudden cardiac death (SCD). Although, in its original description, the disease was believed to predominantly involve the right ventricle, biventricular and left-dominant variants, in which the myocardial lesions affect in parallel or even mostly the left ventricle, are nowadays commonly observed. The clinical management of these patients has two main purposes: the prevention of SCD and the control of arrhythmic and heart failure (HF) events. An implantable cardioverter defibrillator (ICD) is the only proven lifesaving treatment, despite significant morbidity because of device-related complications and inappropriate shocks. Selection of patients who can benefit the most from ICD therapy is one of the most challenging issues in clinical practice. Risk stratification in ACM patients is mostly based on arrhythmic burden and ventricular dysfunction severity, although other clinical features resulting from electrocardiogram and imaging modalities such as cardiac magnetic resonance may have a role. Medical therapy is crucial for treatment of VAs and the prevention of negative ventricular remodelling. In this regard, the efficacy of novel anti-HF molecules and drugs acting on the inflammatory pathway in patients with ACM is, to date, unknown. Catheter ablation represents an effective strategy to treat ventricular tachycardia relapses and recurrent ICD shocks. The present review will address the current strategies for prevention of SCD and treatment of VAs and HF in patients with ACM.

## 1. Introduction

### 1.1. Definition and Classification

Arrhythmogenic cardiomyopathy (ACM) is a genetically determined heart muscle disease characterised pathologically by fibrofatty replacement of right and left ventricular myocardium and clinically by ventricular arrhythmias (VAs) and arrhythmic sudden cardiac death (SCD) [1].

**Figure 1:**
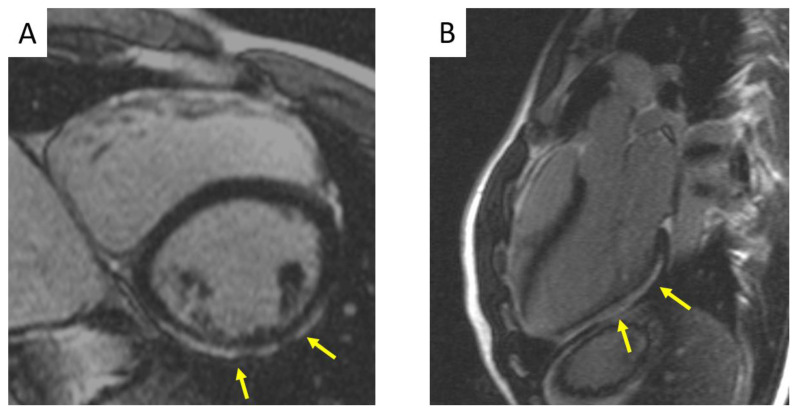
Cardiac magnetic resonance in a patient with a desmosomal gene related biventricular ACM showing the typical LV LGE pattern. (**A**) Short axis view demonstrating subepicardial LGE at the LV mid-inferolateral segments. (**B**) Three-chamber view exhibiting extensive LGE stria at the LV posterolateral wall. ACM = arrhythmogenic cardiomyopathy; LGE = late gadolinium enhancement; LV = left ventricle.

Although the fibrofatty tissue is usually considered the hallmark lesion of ACM, it should be more properly regarded as a marker of advanced stages of the disease [2]. Experimental studies on transgenic animal models showed a histologic pattern consistent with acute myocarditis in the early stages of the disease [3]. The pathological process progresses from the epicardium to the endocardium, leading to wall thinning and aneurysm formation, typically localised at the inferior wall, apex, and infundibulum of the right ventricle (RV) (the so called “triangle of dysplasia”) [2,4]. Indeed, in its original description, the disease was characterised by a predominant involvement of the RV (“Arrhythmogenic right ventricular cardiomyopathy, ARVC”), with left ventricular deterioration occurring later in the history of the disease. However, autopsy investigations, genotype–phenotype correlation studies, and the increasing use of contrast-enhanced cardiac magnetic resonance (CMR) led to the discovery of biventricular and left-dominant variants, in which the myocardial lesions do not remain confined to the RV, but affect in parallel or even predominantly the left ventricle (LV) [5]. When the LV, whose wall is thicker than that of the RV, is involved in the disease process, the fibrofatty scar tends to remain confined to the subepicardial layers, sparing the sub-endocardium, which mostly contribute to myocardial thickening [6]. Indeed, LV lesions do not determine wall thinning nor wall motion abnormalities, making the diagnosis more challenging (Figure 1).

The disease is phenotypically classified in three variants: “right dominant”, characterised by the predominant RV involvement, with no LV abnormalities; “biventricular” with involvement of both the RV and LV; and “left dominant” (also referred to as “Arrhythmogenic left ventricular cardiomyopathy, ALVC”) characterised by a predominant LV involvement, with no RV abnormalities. The broader term “Arrhythmogenic cardiomyopathy, ACM” is currently used to encompass the whole spectrum of the abovementioned disease phenotypic expressions [7]. This term should not be confused with the one of “arrhythmogenic cardiomyopathies”, which has been proposed to comprise a series of different conditions that share non-ischemic myocardial scarring and the propensity to scar-related VAs [7]. In the present review, the term ACM refers to the phenotypic variants of a genetically determined cardiomyopathy whose hallmark lesion is the fibrofatty replacement, which can localise in the RV (ARVC), LV (ALVC) or both ventricles (biventricular ACM).

### 1.2. Genetic Background

Arrhythmogenic cardiomyopathy is generally transmitted as an autosomal dominant trait that is age-related, with incomplete penetrance and variable expressivity. For the classic right-dominant variant, the mutant genes are those encoding for desmosomal proteins, such as plakoglobin (*JUP*), plakophilin-2 (*PKP2*), desmoplakin (*DSP*), desmoglein (*DSG2*), and desmocollin (*DSC2*) [8,9,10,11,12]. In addition, genes encoding for adherent junctional proteins, such as α-T-catenin (*CTNNA3*) and N-cadherin (*CDH2*), have also arose as potentially relevant in the pathogenesis of ACM [13,14]. Desmosomes and adherens junctions provide cellular–mechanical integration, the sodium channels facilitate the initiation of the electrical impulse, and gap junctions mediate the impulse propagations. Altogether, these protein complexes compose structures known as intercalated discs, which ultimately interconnect cardiomyocytes to each other, being responsible for both intercellular electromechanical connections and intracellular signalling cascades (Wnt-β catenin signalling pathway) [15]. A disturbed desmosomal organisation ends in cell death and scarring. However, VAs and SCD can occur before the overt disease [16], as a consequence of purely electrical changes. Indeed, because of a mutation-induced gap junction remodelling, the sodium current can be reduced and cause polymorphic VAs by a mechanism similar to that observed in Brugada Syndrome [17]. Besides the mentioned genes, biventricular and left-dominant forms are usually associated with mutations in non-desmosomal genes encoding for transmembrane protein 43 (*TMEM 43*), lamin A/C (*LMNA*), desmin (*DES*), filamin C (*FLNC*), titin (*TTN*), sodium voltage-gated channel alpha subunit 5 (*SCN5A*), phospholamban (*PLN*), the cardiac ryanodine receptor-2 (*RYR2*) and transforming growth factor beta-3 (*TGFβ-3*) [18,19,20,21,22,23]. Genotyping is not only useful for diagnostic purposes, but also for prognostic reasons. Indeed, some mutant genes in ACM have been associated with a higher risk of SCD and heart failure (HF). In particular, mutations of *TMEM43* p.S358L are characterised by higher disease penetrance and risk of SCD. *FLNC, DES* and *PLN* mutations have been associated with peculiar patterns of LV fibrosis and arrhythmic propensity, with higher risk of VAs and SCD [24,25,26,27,28,29,30,31].

### 1.3. Role of Inflammation in Arrhythmogenic Cardiomyopathy

The presence of inflammatory infiltrates (mainly T-cells) among dying myocytes has been demonstrated in histopathologic analysis of ventricular myocardium at postmortem or in experimental studies on transgenic animals, raising questions about the role of the immune system in the pathogenesis of the disease [2,3]. Arrhythmogenic cardiomyopathy in its early stages may present with acute chest pain and troponins release (“hot phase”), which resembles the infarct-like manifestation of some clinically suspected myocarditis [2,32,33,34]. The presence of autoantibodies has been reported in ACM patients and in their relatives, with a positive status being more frequent in a familial than in a sporadic pattern [35,36]. Whether inflammation is the cause or a consequence of cardiomyocytes apoptosis in ACM remains to be established. The increasing interest in the role of the immune system in the pathogenesis of the disease has translated into pharmacologic research targeting the biologic pathway involved in myocardial inflammation.

### 1.4. Diagnosis

It is important to underline that in ACM the diagnosis is multiparametric. In 1994, an International Task Force (TF) developed the first diagnostic scoring system for the disease, consisting of major and minor criteria grouped into different categories. A “definite” ACM diagnosis was made when multiple criteria (two major criteria, or one major and two minor criteria, or four minor criteria from different categories) were met because no diagnostic test was considered specific enough to reach a final diagnosis. If the number of criteria was not sufficient to satisfy a “Definitive” diagnosis, the disease diagnosis could be downgraded as “borderline” (one major criterion and one minor criterion, or three minor criteria), or “possible” (one major criterion alone, or two minor criteria) [37]. In later years, clinical studies demonstrated that these criteria were highly specific, but lacked sensitivity in mild forms of the disease (for example, in the early diagnosis of family members). Consequently, the revised 2010 TF criteria included new electrocardiographic parameters and quantitative measurements in echocardiogram and CMR imaging to increase this scarce diagnostic sensitivity. Notably, the demonstration of a pathogenic variant in ACM-related genes became a major diagnostic criterion [38]. However, the 2010 TF criteria still had limitations. In 2019, an International Expert Report provided an extensive critical review of their clinical performance, pointing out that the main limitation of the 2010 TF criteria was the absence of specific criteria for the diagnosis of ALVC. In particular, tissue characterisation provided by CMR, which allows the identification of the fibrofatty scar at the LV level to increase the diagnostic sensitivity, was not included [39]. Starting from 2010, there has been growing knowledge of biventricular and left-dominant variants, and in 2017, the term “Arrhythmogenic Cardiomyopathy” was used to give a new definition of the disease [1]. In this context, the 2020 International Expert consensus document provided upgraded criteria (“the Padua Criteria”) for the diagnosis of the entire spectrum of ACM phenotypes, especially the left-sided forms, emphasising the use of myocardial tissue characterisation by CMR for diagnosis [40,41].

## 2. Management

Sudden cardiac death, due to ventricular electrical instability, and HF, as a result of progressive ventricular dilatation and dysfunction, represent the most feared outcomes in ACM. The main objectives in ACM management are: the management of VAs, the prevention of SCD, the attenuation of arrhythmic and HF symptoms, and the slowdown of disease progression. To reach these goals, the patient’s risk profile and symptoms must be assessed, and the most appropriate therapy should be chosen accordingly.

**Figure 2:**
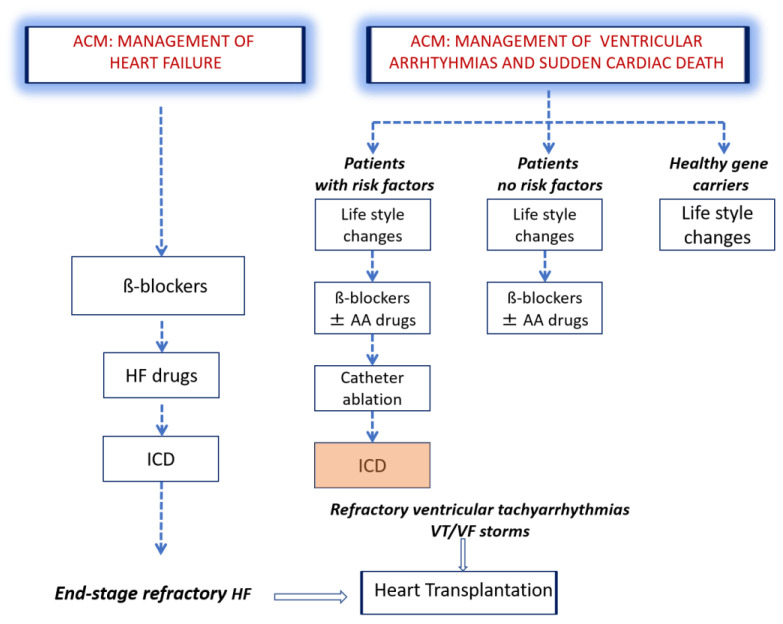
Flow chart of treatment of patients with ACM. Both healthy gene carriers and ACM patients should avoid intense sport activity, to prevent exercise-induced arrhythmic events and disease development or progression. Betablockers, since they prevent arrhythmic events and lower right ventricular wall stress, are essential drugs to be used in all clinically affected individuals. In patients suffering from VAs, AA drugs give the opportunity to improve symptoms. Catheter ablation is an interventional option for patients with episodes of sustained monomorphic VT. Patients for whom the implantation of an ICD is most often indicated are those with history of VF or sustained VT. In advanced stages of the disease, when HF occurs, betablockers and other HF drugs are indicated. AA drugs = antiarrhythmic drugs; ACM = arrhythmogenic cardiomyopathy; HF = heart failure; ICD = implantable cardioverter defibrillator; Vas = ventricular arrhythmias; VT = ventricular tachycardia; VF = ventricular fibrillation.

A flow chart of the treatment of patients with ACM is reported in Figure 2.

### 2.1. Prevention of Sudden Cardiac Death

#### 2.1.1. Risk Stratification

Because ACM patients are often young with a life expectancy of many years, it is of utmost importance to decide whether the patient’s risk of SCD is sufficiently high to justify aggressive therapy, including the insertion of an implantable cardioverter defibrillator (ICD).

Risk stratification in ACM patients is mostly based on the severity of arrhythmias and ventricular dysfunction. Several clinical predictors of poor outcome have been described over the years. Independent predictors of poor prognosis, found in at least one published multivariable analysis, referred as “major” risk factors, are the following: malignant arrhythmic events including SCD, cardiac arrest (CA) due to ventricular tachycardia (VT)/fibrillation (VF), appropriate ICD interventions, or ICD therapy on fast VT/VF; unexplained syncope; non-sustained VT on 24-h Holter monitoring; and RV/LV systolic dysfunction, either severe (RV fractional area change ≤17% or RV EF ≤ 35% for the RV and LV EF ≤ 35% for the LV) or moderate (RV fractional area change between 24 and 17% or RV EF between 40 and 36% for the RV and LV EF between 45 and 36% for the LV). “Minor” risk factors associated with adverse events include: male gender; compound genotype; young age at the time of diagnosis; proband status; inducible VT/VF at programmed ventricular stimulation (PVS); extent of electroanatomic scar and fragmented electrograms on RV voltage mapping; extent of T-wave inversion across precordial and inferior leads; low QRS amplitude and QRS fragmentation, [42]. Some genetic defects have also been associated with a worse arrhythmic outcome. The *TMEM43* p.S358L founder mutation has an almost complete disease penetrance and a high risk of SCD among male carriers [18]. Digenic and compound genotypes are independent predictors of life-threatening VAs and SCD [43]. 

**Figure 3:**
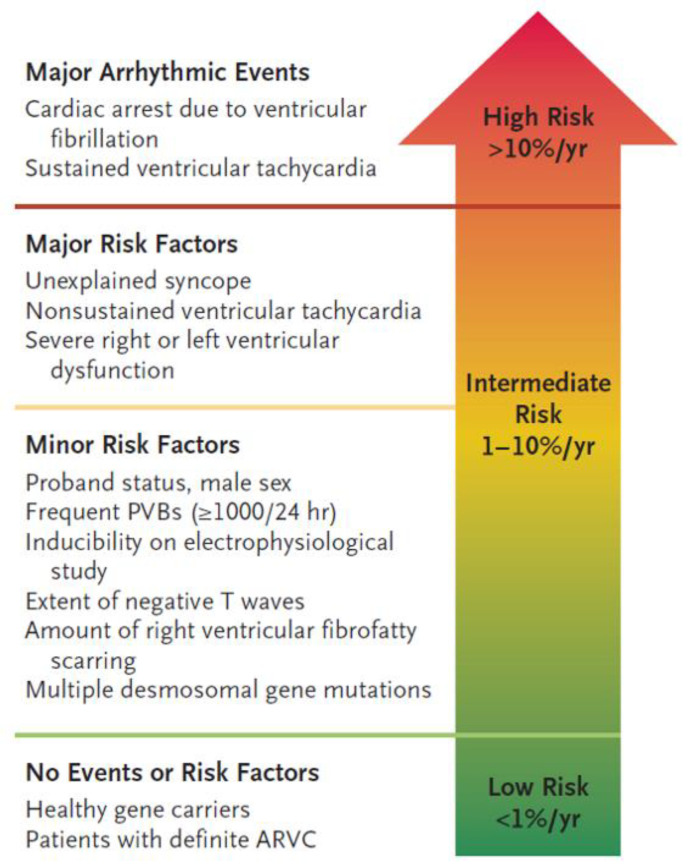
Risk stratification in patients affected by ACM. Risk of major arrhythmic events is based on previous events and specific risk factors. ACM= arrhythmogenic cardiomyopathy. From The New England Journal of Medicine, Domenico Corrado, Mark S. Link, Hugh Calkins, Arrhythmogenic Right Ventricular Cardiomyopathy. New Engl. J. Med. 2017, 376, 61–72. Copyright © 2021 Massachusetts Medical Society. Reprinted with permission [44].

Three categories of arrhythmic risk (“high”, “moderate” and “low” risk) have been identified by the 2015 Task Force consensus document on treatment of ACM (Figure 3). The “high-risk” category comprises patients with a history of CA or hemodynamically unstable VT or those with severe ventricular dysfunction, either right (RV fractional area change ≤17% or RV ejection fraction ≤35%) or left (LV ejection fraction ≤35%). The “intermediate risk” category includes patients with ≥1 “major” risk factors, such as syncope, non-sustained VT, or moderate right (RV fractional area change 17–24% or RV ejection fraction 36–40%) and/or left (LV ejection fraction 36–45%) ventricular dysfunction and patients with ≥1 “minor” risk factors. Asymptomatic patients with no risk factors and healthy gene carriers have a low risk of malignant VAs (“low-risk” category) [42]. Figure 3 illustrates risk categories in patients affected by ACM [44].

Recently, a calculator has been proposed for “primary” risk stratification of patients with ACM [45]. However, this calculator shows significant limitations due to the important selection biases and inhomogeneous study population of the original Cadrin-Tourigny investigation used to predict outcome. Indeed, the outcome predictors were the same factors, such as syncope, non-sustained ventricular VT, and RV (but no LV) systolic dysfunction, which led to ICD implantation. The arrhythmic outcome was assessed using a combined endpoint including appropriate ICD intervention on VT, which is a poor surrogate of SCD because the majority of episodes in ACM patients are self-terminating and even short episodes of fast (>180/min) VT are hemodynamically well tolerated and most often asymptomatic, because the systolic function of the LV is usually preserved or slightly depressed. Since only one-fourth of the total study population had an ICD, 60% of the study patients (without an ICD) were prevented from experiencing an appropriate ICD intervention, which accounted for 70% of outcomes during the follow-up.

The same authors recently developed a new calculator for the prediction of life-threatening VAs (i.e., fast VT/VF, or sudden CA) using the same study design of the previous study [46]. Surprisingly, classic major risk factors such as a history of sustained VT or VF and the severity of ventricular systolic dysfunction did not predict the occurrence of life-threatening VAs. Conversely, malignant arrhythmic events were associated with younger age, male sex, the burden of ectopic ventricular beats and the extent of T-wave inversion in the inferior and precordial leads. The use of the calculator may be associated with overestimation of the risk of VT and VF, which may translate into overtreatment with ICD of asymptomatic ACM patients. Hence, before this calculator can be recommended for clinical use, validation studies are needed to confirm its predictive accuracy among the “real world” ACM patient population (www.arvcrisk.com; accessed on 21 June 2021).

#### 2.1.2. New Risk Predictors

Novel biomarkers are currently emerging as useful tools for risk prediction [47]. Testosterone, plasma bridging integrator 1, soluble ST2, miRNAs, anti-DSG2 antibodies, correlate with disease severity and arrhythmias incidence [35,48,49,50,51,52,53,54,55,56,57,58,59,60]. Rearrangement at the intercalated disk (remodelling of connexin 43 gap junction proteins, sodium channels, and desmosomal proteins, particularly plakoglobin) determined by immunohistochemistry may predict arrhythmic events [61]. Ajmaline challenge can show ST elevation, which is also indicative of sodium channel remodelling [62,63]. Conduction delays, undetectable on 12-lead ECG and associated with a higher risk of arrhythmias, can be unmasked by cardiac activation imaging [64] or by echocardiography deformation imaging [65]. Contrast-enhanced CMR with late gadolinium enhancement (LGE) technique can provide a non-invasive assessment of myocardial fibrosis [66].

#### 2.1.3. Indications for ICD Implantation

**Figure 4:**
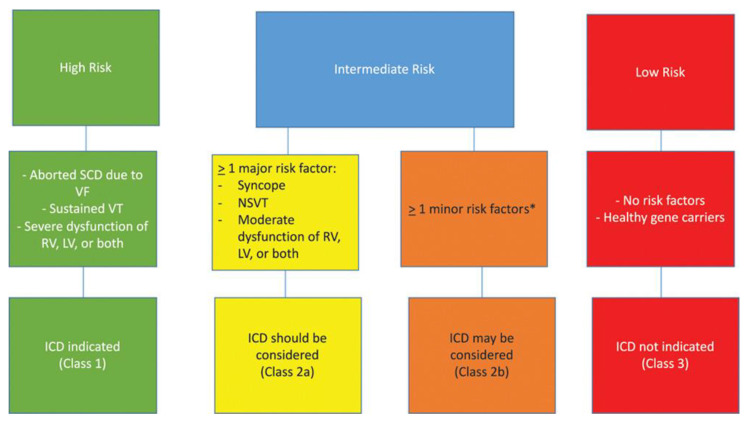
Flow chart of risk stratification and indications of ICD implantation in ACM. Based on the available data on annual mortality rates associated with specific risk factors, the estimated risk of major arrhythmic events in the high-risk category is >10%/year; in the intermediate category, it ranges from 1 to 10%/year; and in the low-risk category, <1%/year. Indications of ICD implantation were determined by consensus considering not only the statistical risk, but also general health, socioeconomic factors, the psychological impact and the adverse effects of the device. * See the text for distinction between major and minor risk factors. ACM = arrhythmogenic cardiomyopathy; ICD = implantable cardioverter defibrillator; LV = left ventricle; NSVT = non sustained ventricular tachycardia; RV = right ventricle; SCD = sudden cardiac death; VF = ventricular fibrillation; VT = ventricular tachycardia. Modified with permission from Corrado et al. [42].

Although randomised trials of ICD therapy have not been performed, data from observational studies have consistently shown that it is effective and safe. The 2015 TF consensus conference on treatment of ACM provided recommendations for ICD implantation with the aim to optimise the prevention of SCD and avoid overtreatment of patients at low risk (Figure 4). Patients who benefit most from ICD are those who have had an episode of VF or sustained VT. It remains uncertain whether ICD therapy is appropriate for primary prevention of SCD among patients with one or more risk factors and no prior major arrhythmic events [63,64].

In asymptomatic patients with no risk factors and in healthy gene carriers, there is generally no indication of prophylactic ICD implantation because of the low risk of arrhythmias and the significant risk of device- and electrode-related complications during long-term follow-up [46].

#### 2.1.4. Transvenous Versus Subcutaneous ICD

ACM patients are usually young and receive an ICD for primary prevention. Thus, it is of utmost importance to prevent SCD whilst avoiding complications such as lead failure, device infections, and inappropriate shocks, frequently associated with the transvenous ICD (TV-ICD) (estimated rate—3.7% per year).

**Figure 5:**
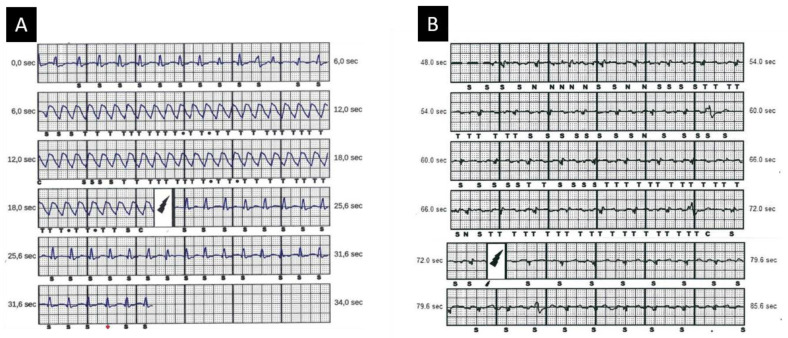
Appropriate and inappropriate S-ICD shocks. (**A**) S-ICD stored electrogram showing appropriately detected and treated ventricular arrhythmia episode. (**B**) S-ICD stored electrogram of inappropriate shock due to P/T-wave oversensing during effort. S-ICD = subcutaneous implantable cardioverter defibrillator. Modified with permission from Migliore et al. [68].

Subcutaneous ICD (S-ICD), thanks to its entirely subcutaneous position, is progressively establishing itself in clinical practice as a valid alternative to the TV-ICD, especially among patients with limited vascular access, increased risk of infection and a structurally normal heart with no need for pacing [67]. In ACM patients, the matter of its use has been more complicated because of the intrinsic characteristics of the disease, which is progressive and associated with the possibility of electrocardiographic depolarisation/repolarisation changes, leading to double QRS counting and P- or T-wave oversensing and potential inappropriate shock delivery [68]. Migliore et al., in a multicentre study enrolling ACM patients receiving S-ICD, demonstrated that S-ICD was a safe and effective therapy for treatment of both induced and spontaneous VAs (Figure 5). However, the use of S-ICD in this population still has some limitations [68]. First, S-ICD cannot deliver anti-tachycardia pacing (ATP) therapy, which may be a “pain-free” solution for VT. However, it has been demonstrated that many of these episodes are self-limiting and haemodynamically well tolerated, thus not needing interruption [69]. Moreover, while re-entrant VTs characterise the advanced stages of the disease, young patients usually suffer VF episodes, reflecting the acute electrical instability of the early phases of the disease [16]. The second issue relies on the higher incidence of inappropriate shocks in ACM patients, which can be triggered by the type of population, mostly of young and active patients, and the higher prevalence of electrocardiographic abnormalities. The latter include: reduced QRS voltages amplitude; negative T-waves; right atrial enlargement with peaked P waves; repolarisation abnormalities depending on the heart rate [69]; and R-wave amplitude decline during follow-up. These electrocardiographic features predispose this population to possible cardiac and/or non-cardiac oversensing and subsequent inappropriate therapy. This is why strategies which aim to reduce inappropriate shocks are based on careful electrocardiographic screening [70], device programming (single vs. dual-zone programming), new implantation techniques [71], and software upgrades such as “SMART Pass” to reduce oversensing [72].

### 2.2. Improvement of Symptoms and Quality of Life

The implant of an ICD is the only therapeutic option of proven efficacy for the prevention of SCD by interruption of otherwise lethal VAs. However, ACM patients often complain of arrhythmic (palpitations, VT recurrences, or ICD discharges) and HF symptoms that affect their quality of life. Currently available treatment options include either pharmacologic or non-pharmacologic therapy.

#### 2.2.1. Traditional Pharmacologic Therapy

Traditional pharmacological therapy includes the use of betablockers, antiarrhythmic drugs (AADs), and HF drugs.

Adrenergic stimulation in ACM patients promotes VAs and SCD, which typically occur during or soon after a physical effort. By preventing effort-induced VAs, betablockers find their ideal location in this setting. Indeed, they receive a class I recommendation for ACM patients symptomatic for frequent premature ventricular beats (PVBs) and non-sustained VT, patients with recurrent VT, appropriate ICD therapies, or inappropriate ICD interventions resulting from sinus tachycardia, supraventricular tachycardia, or atrial fibrillation/flutter with a high ventricular rate. Moreover, they reduce the ventricular wall stress, lowering disease progression. Therefore, there is a class IIa for recommendation of their use in all patients with a definite diagnosis of ACM, irrespective of arrhythmias. Instead, in phenotype-negative gene carriers, prophylactic use of these drugs is not justified [42,73].

Among AADs, amiodarone and sotalol are the most effective drugs with a relatively low proarrhythmic risk. They are recommended in ACM patients with frequent appropriate ICD discharges (class I) to improve symptoms in patients with frequent PVBs and/or non-sustained VT (class IIa) and as an adjunctive therapy when betablockers alone are not sufficient to control the arrhythmic burden in symptomatic patients with frequent PVBs and/or non-sustained VT [42,73].

When right and/or left HF occurs, the standard HF pharmacological treatment including angiotensin-converting enzyme inhibitors, angiotensin II receptor blockers, (betablockers), and diuretics is recommended (class I) [42].

Left-ventricle assist devices and heart transplantation represent the final treatment options when the disease progresses to an end-stage phase. The largest assembled cohort of ACM patients undergoing heart transplant showed high post-transplantation survival rates (94% and 88% at 1 and 6 years, respectively) [74,75,76].

Another not uncommon issue in severely dilated ventricles with aneurysms and sacculations is represented by thrombus formation and related thromboembolic complications. There is no indication for primary prevention with anticoagulants; however, when a thrombus is documented or a thromboembolic event occurs, long-term anticoagulants should be started (class I) [42].

#### 2.2.2. New Pharmacological Options

##### Heart Failure Drugs

The PARADIGM-HF study established the favourable impact of therapy with sacubitril/valsartan (LCZ696) on the outcomes of patients with HF [77]. Whether this treatment is effective in ACM patients with RV, LV or biventricular systolic dysfunction remains to be established. Studies validating the role of these neurohormonal antagonists in ACM represent a major research priority.

##### Anti-Inflammatory Drugs

Glycogen synthase kinase-3β (GSK3 β) and the nuclear factor-kB (NFkB) signalling pathways are abnormally activated in cardiac myocytes in ACM. A small molecule, SB216763 (SB2), a GSK3β inhibitor, appears to prevent or reverse the disease in animal models by reducing the Wtn-β catenin signalling pathway (which is enhanced by GSK3β) [78,79]. However, long-term use of GSK3β antagonists and its effects on the Wtn-β catenin signalling pathway carry an unacceptable risk of developing cancer, limiting their clinical applicability. Thus, subsequent research focused on a downstream level and on inflammatory signalling. NFκB promotes inflammatory responses and interacts with GSK3β. Bay 11-7082, an inhibitor of NFκB, has been demonstrated to reduce the development of disease features [80]. On the same line, the inhibition of the TNFα, IL-1 and NLRP3 inflammasome in experimental models has been shown to have a potential benefit in ACM treatment by reducing inflammation and fibrosis [81,82,83]. These findings gave evidence of the efficacy of targeting the inflammatory pathway as a potential therapeutic option in ACM patients.

Another signalling pathway that is altered, causing LMNA-associated cardiomyopathy, is that of MAP kinase (p38α branch). ARRY-797, a p38 MAPK inhibitor, has been shown to reverse cardiac dysfunction in animal models of *LMNA* and important results are expected from a clinical trial (ClinicalTrials.gov Identifier: NCT03439514) currently underway in patients with dilated cardiomyopathy due to *LMNA* mutations [84].

#### 2.2.3. Non-Pharmacologic Therapy by Catheter Ablation

When pharmacological therapy confers only a partial control of arrhythmias or are poorly tolerated, catheter ablation can represent a potentially effective treatment for recurrent sustained VT episodes and ICD shocks in ACM patients. Therefore, it is recommended in cases of incessant VT or frequent appropriate ICD interventions on VT, despite maximal pharmacological therapy, and may be considered in patients who do not desire or cannot tolerate pharmacological therapies [42]. Since catheter ablation does not prevent SCD, it should not be considered as an alternative to ICD implantation in ACM patients with a history of sustained VT, with the possible exception of selected cases with a drug refractory, haemodynamically stable, single morphology VT [42].

Myocardial scars are imaged as low-voltage regions (areas ≥1 cm^2^ with low bipolar voltages (<1.5 mV), and fractionated potentials (i.e., >3 deflections, amplitude ≤1.5 mV, duration >70 ms)) by bipolar three-dimensional electroanatomic voltage mapping (3D-EVM). As mentioned above, these alterations not only have a diagnostic value but also carry a prognostic significance as they correlate with arrhythmic events. However, 3D-EVM can miss low-voltage areas in about 25–30% of cases and the success rate of the endocardial approach is still modest because of the epicardial location of the majority of VT circuits [85]. This epicardial substrate can be evidenced as abnormal epicardial bipolar low-voltage areas (amplitude < 1.0 mV) by epicardial voltage mapping [86] or, as an alternative to this invasive approach, by using a threshold of <5.5 mV at unipolar endocardial mapping [87].

**Figure 6:**
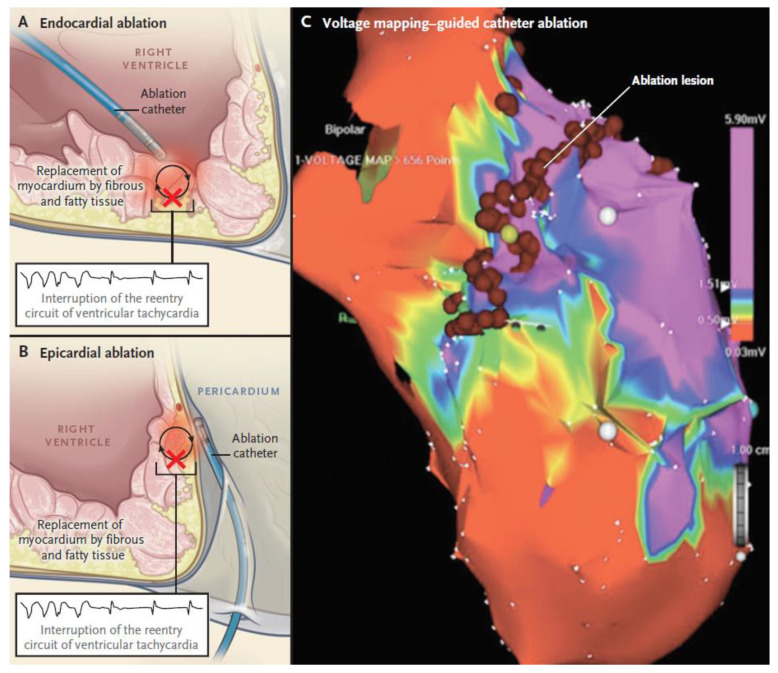
Catheter ablation in ACM patients. Panel (**A**) shows the subendocardial approach. Panel (**B**) shows the subepicardial approach. Panel (**C**) shows three-dimensional electro-anatomical voltage mapping to reconstruct regions of right ventricular scarring. ACM = arrhythmogenic cardiomyopathy. From The New England Journal of Medicine, Domenico Corrado, Mark S. Link, Hugh Calkins, Arrhythmogenic Right Ventricular Cardiomyopathy. New Engl. J. Med. 2017, 376, 61–72. Copyright © 2021 Massachusetts Medical Society. Reprinted with permission [44].

The catheter ablation technique includes patient sedation, mapping, electrophysiological study, and ablation itself. For the endocardial approach, conscious sedation is favoured, while for the epicardial approach, general anaesthesia is preferred. The endocardial approach relies on a standard transfemoral access, whereas a pericardial access is employed in the epicardial approach (Figure 6). The following steps are similar for both the approaches: use of irrigated-tip catheters with contact force sensors to detect fractionated signals and late potentials, pace mapping to identify additional sites, PVS to induce VT, activation, and entrainment VT mapping to identify the tachycardia circuit or, as an alternative, a substrate-based ablation targeting “channels” and delayed/fractioned potentials within low voltages areas.

Patients with end-stage ACM require more extensive radiofrequency applications be delivered at the endocardium because of a wider endocardial than epicardial involvement. However, even in these cases, the epicardial approach (combined with endocardial) may be useful to achieve complete substrate elimination and decrease VT episodes during follow-up. Because recent studies demonstrated good long-term outcomes in patients showing no VT inducibility after endocardial-only ablation, the epicardial approach should be reserved only for when spontaneous or inducible VT persists after extensive previously performed endocardial ablation [88,89] (Table 1). Therefore, a stepwise method with a first attempt with endocardial-only ablation followed eventually by epicardial ablation in those patients exhibiting a bipolar vs. unipolar low-voltage areas may be preferred.

## 3. Conclusions

Arrhythmogenic cardiomyopathy is characterised by progressive scarring of the ventricular myocardium and ventricular dilatation and dysfunction.

The clinical approach to the disease should embrace new concepts and awareness regarding the pathobiological basis and the role of the immune system in the development and progression of the disease. Risk stratification to guide ICD implantation remains a crucial point in the management of ACM patients for primary prevention of SCD. The emerging role of S-ICD offers the potential to revise the indications of ICD treatment. The significant advances of mapping and catheter ablation have led to effective non-pharmacologic therapy of sustained VT. Neurohormonal antagonists and drugs targeting the Wtn-β and NFκB pathways represent the major advances of pharmacological treatment.

## Figures and Tables

**Table 1:** Major series of ventricular tachycardia ablation outcomes in arrhythmogenic cardiomyopathy.

Author (Year)	Patients n (Men)	Ablation Technique			Complete Acute Success (%)	Procedure-Related Complications	Follow-up		
		Electro- Anatomic Map	Irrigated Tip	Epicardial Map/abl (%)			Mean (Months)	VT Recurrences (%)	Deaths or HT
Santangeli 2019	32 (23)	Yes	Yes	72%	100	1 (RV laceration)	46	19	N/A
Berruezo 2017	41 (36)	Yes	Yes	100%	90	2 (tamponade, death)	32	26.8	N/A
Mussigbrodt 2017	45 (30)	Yes	Yes	48.9%	84	5 (TIA, tamponade x2, PE x2 1 fatal)	31	44 *	N/A
Souissi 2018	49 (44)	Yes	Yes	100%	71	3 (tamponade, femoral AV fistula, intestinal perforation)	64	81 at 5 years 31 at 1 years *	6 deaths, 2 HT
Santangeli 2015	62 (45)	Yes	Yes	63%	77	5 (PE x2, pericardial effusion, RV puncture, CT)	56	29 *	5 NC, 5HT
Philips 2012	87 (45)	Yes	Yes	26.4%	82	2 (death, MI)	88	85	N/A
Berruezo 2012	11 (9)	Yes	Yes	100%	100	1 (tamponade)	11	9	0
Garcia 2009	13 (10)	Yes	Yes	Yes	92	0	18	23	1 HT
Nogami 2008	18 (13)	Yes	No	No	72	0	61	33	2 HF, 1 NC
Dalal, 2007	24 (11)	Yes	No	No	77	1 (death)	32	85	2 HT
Satomi 2006	17 (13)	Yes	No	No	88	0	26	24	0
Verma 2005	22 (15)	Yes	Yes	No	82	1 (tamponade)	37	36	0
Miljoen 2005	11 (8)	Yes	No	No	73	0	20	45	1 NC
Marchlinski 2004	19 (18)	Yes	Yes	No	74	0	27	11	0
Reithmann 2003	5 (3)	Yes	No	No	80	0	7	20	0
Ellison 1998	5 (4)	No	No	No	42	0	17	0	0

* The endpoint was freedom from ventricular tachycardia after the last ablation. AV = arterovenous, CT = constrictive pericarditis, HF = heart failure death, HT = heart transplantation, MI = myocardial infarction, NC = non-cardiac death, PE = pulmonary embolism, TIA = transient ischemic attack, RV = right ventricular.

## Data Availability

Data available in a publicly accessible repository.

## References

[1] Corrado D., Basso C., Judge D. (2017). Arrhythmogenic Cardiomyopathy. Circ. Res..

[2] Basso C., Thiene G., Corrado D., Angelini A., Nava A., Valente M. (1996). Arrhythmogenic Right Ventricular Cardiomyopathy: Dysplasia, dystrophy, or myocarditis?. Circulation.

[3] Pilichou K., Remme C.A., Basso C., Campian M.E., Rizzo S., Barnett P., Scicluna B., Bauce B., Hoff M.J.B.V.D., De Bakker J.M.T. (2009). Myocyte necrosis underlies progressive myocardial dystrophy in mouse dsg2-related arrhythmogenic right ventricular cardiomyopathy. J. Exp. Med..

[4] Thiene G., Basso C. (2001). Arrhythmogenic right ventricular cardiomyopathy: An update. Cardiovasc. Pathol..

[5] Sen-Chowdhry S., Syrris P., Prasad S.K., Hughes S.E., Merrifield R., Ward D., Pennell D., McKenna W.J. (2008). Left-Dominant Arrhythmogenic Cardiomyopathy. J. Am. Coll. Cardiol..

[6] Te Riele A.S.J.M., James C.A., Philips B., Rastegar N., Bhonsale A., Groeneweg J.A., Murray B., Tichnell C., Judge D., Van Der Heijden J.F. (2013). Mutation-Positive Arrhythmogenic Right Ventricular Dysplasia/Cardiomyopathy: The Triangle of Dysplasia Displaced. J. Cardiovasc. Electrophysiol..

[7] Corrado D., Van Tintelen P.J., McKenna W.J., Hauer R.N.W., Anastastakis A., Asimaki A., Basso C., Bauce B., Brunckhorst C., Bucciarelli-Ducci C. (2020). International Experts. Arrhythmogenic right ventricular cardiomyopathy: Evaluation of the current diagnostic criteria and differential diagnosis. Eur. Heart J..

[8] McKoy G., Protonotarios N., Crosby A., Tsatsopoulou A., Anastasakis A., Coonar A., Norman M., Baboonian C., Jeffery S., McKenna W.J. (2000). Identification of a deletion in plakoglobin in arrhythmogenic right ventricular cardiomyopathy with palmoplantar keratoderma and woolly hair (Naxos disease). Lancet.

[9] Rampazzo A., Nava A., Malacrida S., Beffagna G., Bauce B., Rossi V., Zimbello R., Simionati B., Basso C., Thiene G. (2002). Mutation in Human Desmoplakin Domain Binding to Plakoglobin Causes a Dominant Form of Arrhythmogenic Right Ventricular Cardiomyopathy. Am. J. Hum. Genet..

[10] Gerull B., Heuser A., Wichter T., Paul M., Basson C.T., A McDermott D., Lerman B.B., Markowitz S.M., Ellinor P.T., Macrae C.A. (2004). Mutations in the desmosomal protein plakophilin-2 are common in arrhythmogenic right ventricular cardiomyopathy. Nat. Genet..

[11] Pilichou K., Nava A., Basso C., Beffagna G., Bauce B., Lorenzon A., Frigo G., Vettori A., Valente M., Towbin J. (2006). Mutations in Desmoglein-2 Gene Are Associated with Arrhythmogenic Right Ventricular Cardiomyopathy. Circulation.

[12] Syrris P., Ward D., Evans A., Asimaki A., Gandjbakhch E., Sen-Chowdhry S., McKenna W.J. (2006). Arrhythmogenic Right Ventricular Dysplasia/Cardiomyopathy Associated with Mutations in the Desmosomal Gene Desmocollin-2. Am. J. Hum. Genet..

[13] Van Hengel J., Calore M., Bauce B., Dazzo E., Mazzotti E., De Bortoli M., Lorenzon A., Mura I.E.L., Beffagna G., Rigato I. (2013). Mutations in the area composita protein αT-catenin are associated with arrhythmogenic right ventricular cardiomyopathy. Eur. Hear. J..

[14] Mayosi B.M., Fish M., Shaboodien G., Mastantuono E., Kraus S., Wieland T., Kotta M.-C., Chin A., Laing N., Ntusi N.B. (2017). Identification of Cadherin 2 (CDH2) Mutations in Arrhythmogenic Right Ventricular Cardiomyopathy. Circ. Cardiovasc. Genet..

[15] Gras E.G., Lombardi R., Giocondo M.J., Willerson J.T., Schneider M.D., Khoury D.S., Marian A.J. (2006). Suppression of canonical Wnt/ -catenin signaling by nuclear plakoglobin recapitulates phenotype of arrhythmogenic right ventricular cardiomyopathy. J. Clin. Investig..

[16] Mattesi G., Zorzi A., Corrado D., Cipriani A. (2020). Natural History of Arrhythmogenic Cardiomyopathy. J. Clin. Med..

[17] Corrado D., Zorzi A., Cerrone M., Rigato I., Mongillo M., Bauce B., Delmar M. (2016). Relationship Between Arrhythmogenic Right Ventricular Cardiomyopathy and Brugada Syndrome. Circ. Arrhythmia Electrophysiol..

[18] Merner N.D., Hodgkinson K.A., Haywood A.F., Connors S., French V.M., Drenckhahn J.-D., Kupprion C., Ramadanova K., Thierfelder L., McKenna W. (2008). Arrhythmogenic Right Ventricular Cardiomyopathy Type 5 Is a Fully Penetrant, Lethal Arrhythmic Disorder Caused by a Missense Mutation in the TMEM43 Gene. Am. J. Hum. Genet..

[19] Quarta G., Syrris P., Ashworth M., Jenkins S., Alapi K.Z., Morgan J., Muir A., Pantazis A., McKenna W.J., Elliott P.M. (2011). Mutations in the Lamin A/C gene mimic arrhythmogenic right ventricular cardiomyopathy. Eur. Hear. J..

[20] van Tintelen J.P., Van Gelder I.C., Asimaki A., Suurmeijer A.J., Wiesfeld A.C., Jongbloed J.D., Wijngaard A.V.D., Kuks J.B., van Spaendonck-Zwarts K.Y., Notermans N. (2009). Severe cardiac phenotype with right ventricular predominance in a large cohort of patients with a single missense mutation in the DES gene. Hear. Rhythm..

[21] Taylor M., Graw S., Sinagra G., Barnes C., Slavov D., Brun F., Pinamonti B., Salcedo E.E., Sauer W., Pyxaras S. (2011). Genetic Variation in Titin in Arrhythmogenic Right Ventricular Cardiomyopathy–Overlap Syndromes. Circulation.

[22] Van Der Zwaag P.A., Van Rijsingen I.A., Asimaki A., Jongbloed J.D., Van Veldhuisen D.J., Wiesfeld A.C., Cox M.G., Van Lochem L.T., De Boer R.A., Hofstra R.M. (2012). Phospholamban R14del mutation in patients diagnosed with dilated cardiomyopathy or arrhythmogenic right ventricular cardiomyopathy: Evidence supporting the concept of arrhythmogenic cardiomyopathy. Eur. J. Hear. Fail..

[23] Beffagna G., Occhi G., Nava A., Vitiello L., Ditadi A., Basso C., Bauce B., Carraro G., Thiene G., Towbin J.A. (2005). Regulatory mutations in transforming growth factor-?3 gene cause arrhythmogenic right ventricular cardiomyopathy type 1. Cardiovasc. Res..

[24] Sen-Chowdhry S., Syrris P., Ward D., Asimaki A., Sevdalis E., McKenna W.J. (2007). Clinical and Genetic Characterization of Families with Arrhythmogenic Right Ventricular Dysplasia/Cardiomyopathy Provides Novel Insights Into Patterns of Disease Expression. Circulation.

[25] Bhonsale A., Groeneweg J.A., James C.A., Dooijes D., Tichnell C., Jongbloed J.D.H., Murray B., Riele A.S.J.M.T., Berg M.P.V.D., Bikker H. (2015). Impact of genotype on clinical course in arrhythmogenic right ventricular dysplasia/cardiomyopathy-associated mutation carriers. Eur. Hear. J..

[26] Ortiz-Genga M.F., Cuenca S., Ferro M.D., Zorio E., Aranda R.S., Climent V., Padrón-Barthe L., Duro-Aguado I., Jiménez-Jáimez J., Hidalgo-Olivares V.M. (2016). Truncating FLNC Mutations Are Associated With High-Risk Dilated and Arrhythmogenic Cardiomyopathies. J. Am. Coll. Cardiol..

[27] Dominguez F., Zorio E., Jimenez-Jaimez J., Salguero-Bodes R., Zwart R., Gonzalez-Lopez E., Molina P., Jiménez F.J.B., Delgado J.F., Braza-Boïls A. (2020). Clinical characteristics and determinants of the phenotype in TMEM43 arrhythmogenic right ventricular cardiomyopathy type 5. Hear. Rhythm..

[28] Begay R., Graw S.L., Sinagra G., Asimaki A., Rowland T.J., Slavov D.B., Gowan K., Jones K.L., Brun F., Merlo M. (2018). Filamin C Truncation Mutations Are Associated with Arrhythmogenic Dilated Cardiomyopathy and Changes in the Cell–Cell Adhesion Structures. JACC Clin. Electrophysiol..

[29] Augusto J.B., Eiros R., Nakou E., Moura-Ferreira S., Treibel T., Captur G., Akhtar M.M., Protonotarios A., Gossios T.D., Savvatis K. (2019). Dilated cardiomyopathy and arrhythmogenic left ventricular cardiomyopathy: A comprehensive genotype-imaging phenotype study. Eur. Hear. J. Cardiovasc. Imaging.

[30] Segura-Rodríguez D., Jiménez F.J.B., Carriel V., López-Fernández S., González-Molina M., Ramírez J.M.O., Fernández-Navarro L., García-Roa M.D., Cabrerizo E.M., Durand-Herrera D. (2019). Myocardial fibrosis in arrhythmogenic cardiomyopathy: A genotype–phenotype correlation study. Eur. Hear. J. Cardiovasc. Imaging.

[31] Mattesi G., Cipriani A., Bauce B., Rigato I., Zorzi A., Corrado D. (2021). Arrhythmogenic Left Ventricular Cardiomyopathy: Genotype-Phenotype Correlations and New Diagnostic Criteria. J. Clin. Med..

[32] Calabrese F., Angelini A., Thiene G., Basso C., Nava A., Valente M. (2000). No detection of enteroviral genome in the myocardium of patients with arrhythmogenic right ventricular cardiomyopathy. J. Clin. Pathol..

[33] Thiene G., Corrado D., Nava A., Rossi L., Poletti A., Boffa G.M., Daliento L., Pennelli N. (1991). Right ventricular cardiomyopathy: Is there evidence of an inflammatory aetiology?. Eur. Hear. J..

[34] Bariani R., Cipriani A., Rizzo S., Celeghin R., Marinas M.B., Giorgi B., De Gaspari M., Rigato I., Leoni L., Zorzi A. (2021). ‘Hot phase’ clinical presentation in arrhythmogenic cardiomyopathy. Europace.

[35] Chatterjee D., Fatah M., Akdis D., A Spears D., Koopmann T.T., Mittal K., A Rafiq M., Cattanach B.M., Zhao Q., Healey J.S. (2018). An autoantibody identifies arrhythmogenic right ventricular cardiomyopathy and participates in its pathogenesis. Eur. Hear. J..

[36] Caforio A.L., Re F., Avella A., Marcolongo R., Baratta P., Seguso M., Gallo N., Plebani M., Izquierdo-Bajo A., Cheng C.-Y. (2020). Evidence From Family Studies for Autoimmunity in Arrhythmogenic Right Ventricular Cardiomyopathy. Circulation.

[37] McKenna W.J., Thiene G., Nava A., Fontaliran F., Blomstrom-Lundqvist C., Fontaine G., Camerini F. (1994). Diagnosis of arrhythmogenic right ventricular dysplasia/cardiomyopathy. Task Force of the Working Group Myocardial and Pericardial Disease of the European Society of Cardiology and of the Scientific Council on Cardiomyopathies of the International Society and Federation of Cardiology. Br. Heart. J..

[38] Marcus F.I., McKenna W.J., Sherrill D., Basso C., Bauce B., Bluemke D., Calkins H., Corrado D., Cox M.G., Daubert J.P. (2010). Diagnosis of Arrhythmogenic Right Ventricular Cardiomyopathy/Dysplasia. Circulation.

[39] Towbin J.A., McKenna W.J., Abrams D.J., Ackerman M.J., Calkins H., Darrieux F.C., Daubert J.P., De Chillou C., DePasquale E.C., Desai M.Y. (2019). 2019 HRS expert consensus statement on evaluation, risk stratification, and management of arrhythmogenic cardiomyopathy. Hear. Rhythm..

[40] Corrado D., Perazzolo Marra M., Zorzi A., Beffagna G., Cipriani A., Lazzari M., Migliore F., Pilichou K., Rampazzo A., Rigato I. (2020). Diagnosis of arrhythmogenic cardiomyopathy: The Padua criteria. Int. J. Cardiol..

[41] Pontone G., Di Bella G., Castelletti S., Maestrini V., Festa P., Ait-Ali L., Masci P.G., Monti L., Di Giovine G., De Lazzari M. (2017). Clinical recommendations of cardiac magnetic resonance, Part II. J. Cardiovasc. Med..

[42] Corrado D., Wichter T., Link M.S., Hauer R.N., Marchlinski F.E., Anastasakis A., Bauce B., Basso C., Brunckhorst C., Tsatsopoulou A. (2015). Treatment of Arrhythmogenic Right Ventricular Cardiomyopathy/Dysplasia. Circulation.

[43] Rigato I., Bauce B., Rampazzo A., Zorzi A., Pilichou K., Mazzotti E., Migliore F., Marra M.P., Lorenzon A., De Bortoli M. (2013). Compound and Digenic Heterozygosity Predicts Lifetime Arrhythmic Outcome and Sudden Cardiac Death in Desmosomal Gene–Related Arrhythmogenic Right Ventricular Cardiomyopathy. Circ. Cardiovasc. Genet..

[44] Corrado D., Link M.S., Calkins H. (2017). Arrhythmogenic Right Ventricular Cardiomyopathy. New Engl. J. Med..

[45] Cadrin-Tourigny J., Bosman L.P., Nozza A., Wang W., Tadros R., Bhonsale A., Bourfiss M., Fortier A., Lie Ø.H., Saguner A.M. (2019). A new prediction model for ventricular arrhythmias in arrhythmogenic right ventricular cardiomyopathy. Eur. Hear. J..

[46] Cadrin-Tourigny J., Bosman L.P., Wang W., Tadros R., Bhonsale A., Bourfiss M., Lie Ø.H., Saguner A.M., Svensson A., Andorin A. (2021). Sudden Cardiac Death Prediction in Arrhythmogenic Right Ventricular Cardiomyo-pathy: A Multinational Collaboration. Circ. Arrhythm. Electrophysiol..

[47] Van Der Voorn S.M., Riele A.S.J.M.T., Basso C., Calkins H., Remme C.A., Veen T.A.B.V. (2020). Arrhythmogenic cardiomyopathy: Pathogenesis, pro-arrhythmic remodelling, and novel approaches for risk stratification and therapy. Cardiovasc. Res..

[48] Akdis D., Saguner A.M., Shah K., Wei C., Medeiros-Domingo A., Von Eckardstein A., Lüscher T.F., Brunckhorst C., Chen H.V., Duru F. (2017). Sex hormones affect outcome in arrhythmogenic right ventricular cardiomyopathy/dysplasia: From a stem cell derived cardiomyocyte-based model to clinical biomarkers of disease outcome. Eur. Hear. J..

[49] Coats C.J., E Heywood W., Mills K., Elliott P.M. (2015). Current applications of biomarkers in cardiomyopathies. Expert Rev. Cardiovasc. Ther..

[50] De Jong S., Van Veen T.A.B., De Bakker J.M.T., Vos M.A., van Rijen H. (2011). Biomarkers of Myocardial Fibrosis. J. Cardiovasc. Pharmacol..

[51] De Jong S., Van Veen T.A.B., De Bakker J.M.T., Van Rijen H.V.M. (2011). Monitoring cardiac fibrosis: A technical challenge. Neth. Hear. J..

[52] Chalikias G.K., Tziakas D.N. (2015). Biomarkers of the extracellular matrix and of collagen fragments. Clin. Chim. Acta.

[53] Spinale F.G. (2002). Matrix Metalloproteinases. Circ. Res..

[54] Sommariva E., D’Alessandra Y., Farina F.M., Casella M., Cattaneo F., Catto V., Chiesa M., Stadiotti I., Brambilla S., Russo A.D. (2017). MiR-320a as a Potential Novel Circulating Biomarker of Arrhythmogenic CardioMyopathy. Sci. Rep..

[55] Zhang H., Liu S., Dong T., Yang J., Xie Y., Wu Y., Kang K., Hu S., Gou D., Wei Y. (2016). Profiling of differentially expressed microRNAs in arrhythmogenic right ventricular cardiomyopathy. Sci. Rep..

[56] Thum T., Condorelli G. (2015). Long Noncoding RNAs and MicroRNAs in Cardiovascular Pathophysiology. Circ. Res..

[57] Bauersachs J. (2010). Regulation of Myocardial Fibrosis by MicroRNAs. J. Cardiovasc. Pharmacol..

[58] Thum T., Gross C., Fiedler J., Fischer T., Kissler S., Bussen M., Galuppo P., Just S., Rottbauer W., Frantz S. (2008). MicroRNA-21 contributes to myocardial disease by stimulating MAP kinase signalling in fibroblasts. Nat. Cell Biol..

[59] Broch K., Leren I.S., Saberniak J., Ueland T., Edvardsen T., Gullestad L., Haugaa K. (2017). Soluble ST2 is associated with disease severity in arrhythmogenic right ventricular cardiomyopathy. Biomarkers.

[60] Hong T.-T., Cogswell R., James C.A., Kang G., Pullinger C.R., Malloy M.J., Kane J.P., Wojciak J., Calkins H., Scheinman M.M. (2012). Plasma BIN1 correlates with heart failure and predicts arrhythmia in patients with arrhythmogenic right ventricular cardiomyopathy. Hear. Rhythm..

[61] Asimaki A., Protonotarios A., James C.A., Chelko S., Tichnell C., Murray B., Tsatsopoulou A., Anastasakis A., Riele A.T., Kléber A.G. (2016). Characterizing the Molecular Pathology of Arrhythmogenic Cardiomyopathy in Patient Buccal Mucosa Cells. Circ. Arrhythmia Electrophysiol..

[62] Rolf S., Bruns H.-J., Wichter T., Kirchhof P., Ribbing M., Wasmer K., Paul M., Breithardt G., Haverkamp W., Eckardt L. (2003). The ajmaline challenge in Brugada syndrome: Diagnostic impact, safety, and recommended protocol. Eur. Hear. J..

[63] Peters S. (2008). Arrhythmogenic right ventricular dysplasia-cardiomyopathy and provocable coved-type ST-segment elevation in right precordial leads: Clues from long-term follow-up. Europace.

[64] Oostendorp T.F., Van Dessel P.F.H.M., Coronel R., Belterman C., Linnenbank A.C., Van Schie I.H., Van Oosterom A., Oosterhoff P., Van Dam P.M., De Bakker J.M.T. (2011). Noninvasive detection of epicardial and endocardial activity of the heart. Neth. Hear. J..

[65] Teske A.J., Cox M.G., Riele A.T., De Boeck B.W., Doevendans P.A., Hauer R.N., Cramer M.J. (2012). Early Detection of Regional Functional Abnormalities in Asymptomatic ARVD/C Gene Carriers. J. Am. Soc. Echocardiogr..

[66] Haugaa K.H., Haland T.F., Leren I.S., Saberniak J., Edvardsen T. (2015). Arrhythmogenic right ventricular cardiomyopathy, clinical manifestations, and diagnosis. Europace.

[67] Knops R.E., Nordkamp L.R.O., Delnoy P.-P.H., Boersma L.V., Kuschyk J., El-Chami M.F., Bonnemeier H., Behr E.R., Brouwer T.F., Kääb S. (2020). Subcutaneous or Transvenous Defibrillator Therapy. N. Engl. J. Med..

[68] Migliore F., Viani S., Bongiorni M.G., Zorzi A., Silvetti M.S., Francia P., D’Onofrio A., De Franceschi P., Sala S., Donzelli S. (2019). Subcutaneous implantable cardioverter defibrillator in patients with arrhythmogenic right ventricular cardiomyopathy: Results from an Italian multicenter registry. Int. J. Cardiol..

[69] Link M.S., Laidlaw D., Polonsky B., Zareba W., McNitt S., Gear K., Marcus F., Estes N.A.M. (2014). Ventricular Arrhythmias in the North American Multidisciplinary Study of ARVC. J. Am. Coll. Cardiol..

[70] Migliore F., Bertaglia E., Zorzi A., Corrado D. (2017). Subcutaneous Implantable Cardioverter-Defibrillator and Arrhythmogenic Right Ventricular Cardiomyopathy. JACC Clin. Electrophysiol..

[71] Migliore F., Mattesi G., De Franceschi P., Allocca G., Crosato M., Calzolari V., Fantinel M., Ortis B., Facchin D., Daleffe E. (2019). Multicentre experience with the second-generation subcutaneous implantable cardioverter defibrillator and the intermuscular two-incision implantation technique. J. Cardiovasc. Electrophysiol..

[72] Theuns D.A., Brouwer T.F., Jones P.W., Allavatam V., Donnelley S., Auricchio A., Knops R.E., Burke M.C. (2018). Prospective blinded evaluation of a novel sensing methodology designed to reduce inappropriate shocks by the subcutaneous implantable cardioverter-defibrillator. Hear. Rhythm..

[73] Priori S., Blomström-Lundqvist C., Mazzanti A., Blom N., Borggrefe M., Camm J., Elliott P.M., Fitzsimons D., Hatala R., Hindricks G. (2015). 2015 ESC Guidelines for the management of patients with ventricular arrhythmias and the prevention of sudden cardiac death. Eur. Hear. J..

[74] Tedford R.J., James C., Judge D., Tichnell C., Murray B., Bhonsale A., Philips B., Abraham T., Dalal D., Halushka M. (2012). Cardiac Transplantation in Arrhythmogenic Right Ventricular Dysplasia/Cardiomyopathy. J. Am. Coll. Cardiol..

[75] Yancy C.W., Jessup M., Bozkurt B., Butler J., Casey D.E., Drazner M.H., Fonarow G.C., Geraci S.A., Horwich T., Januzzi J.L. (2013). 2013 ACCF/AHA Guideline for the Management of Heart Failure: A report of the American College of Cardiology Foundation/American Heart Association Task Force on Practice Guidelines. J. Am. Coll. Cardiol..

[76] Ponikowski P., Voors A.A., Anker S.D., Bueno H., Cleland J.G.F., Coats A.J.S., Falk V., González-Juanatey J.R., Harjola V.-P., Jankowska E.A. (2016). 2016 ESC Guidelines for the diagnosis and treatment of acute and chronic heart failure: The Task Force for the diagnosis and treatment of acute and chronic heart failure of the European Society of Cardiology (ESC). Developed with the special contribution of the Heart Failure Association (HFA) of the ESC. Eur. J. Heart Fail..

[77] Mogensen U.M., Gong J., Jhund P., Shen L., Køber L., Desai A.S., Lefkowitz M.P., Packer M., Rouleau J.L., Solomon S.D. (2018). Effect of sacubitril/valsartan on recurrent events in the Prospective comparison of ARNI with ACEI to Determine Impact on Global Mortality and morbidity in Heart Failure trial (PARADIGM-HF). Eur. J. Hear. Fail..

[78] Asimaki A., Kapoor S., Plovie E., Arndt A.K., Adams E., Liu Z., James C.A., Judge D., Calkins H., Churko J. (2014). Identification of a New Modulator of the Intercalated Disc in a Zebrafish Model of Arrhythmogenic Cardiomyopathy. Sci. Transl. Med..

[79] Chelko S., Asimaki A., Andersen P., Bedja D., Amat-Alarcon N., DeMazumder D., Jasti R., Macrae C.A., Leber R., Kleber A.G. (2016). Central role for GSK3β in the pathogenesis of arrhythmogenic cardiomyopathy. JCI Insight.

[80] Chelko S., Asimaki A., Lowenthal J., Bueno-Beti C., Bedja D., Scalco A., Amat-Alarcon N., Andersen P., Judge D.P., Tung L. (2019). Therapeutic Modulation of the Immune Response in Arrhythmogenic Cardiomyopathy. Circulation.

[81] Zhang X., Meng F., Song J., Zhang L., Wang J., Li D., Li L., Dong P., Yang B., Chen Y. (2016). Pentoxifylline Ameliorates Cardiac Fibrosis, Pathological Hypertrophy, and Cardiac Dysfunction in Angiotensin II-induced Hypertensive Rats. J. Cardiovasc. Pharmacol..

[82] Szekely Y., Arbel Y. (2018). A Review of Interleukin-1 in Heart Disease: Where Do We Stand Today?. Cardiol. Ther..

[83] Ridker P.M., Everett B.M., Thuren T., MacFadyen J.G., Chang W.H., Ballantyne C., Fonseca F., Nicolau J., Koenig W., Anker S.D. (2017). Antiinflammatory Therapy with Canakinumab for Atherosclerotic Disease. N. Engl. J. Med..

[84] Muchir A., Wu W., Choi J.C., Iwata S., Morrow J., Homma S., Worman H.J. (2012). Abnormal p38 mitogen-activated protein kinase signaling in dilated cardiomyopathy caused by lamin A/C gene mutation. Hum. Mol. Genet..

[85] Philips B., Madhavan S., James C., Tichnell C., Murray B., Dalal D., Bhonsale A., Nazarian S., Judge D., Russell S.D. (2012). Outcomes of Catheter Ablation of Ventricular Tachycardia in Arrhythmogenic Right Ventricular Dysplasia/Cardiomyopathy. Circ. Arrhythmia Electrophysiol..

[86] Garcia F.C., Bazan V., Zado E.S., Ren J.-F., Marchlinski F. (2009). Epicardial Substrate and Outcome with Epicardial Ablation of Ventricular Tachycardia in Arrhythmogenic Right Ventricular Cardiomyopathy/Dysplasia. Circulation.

[87] Polin G.M., Haqqani H., Tzou W., Hutchinson M., Garcia F.C., Callans D.J., Zado E.S., Marchlinski F.E. (2011). Endocardial unipolar voltage mapping to identify epicardial substrate in arrhythmogenic right ventricular cardiomyopathy/dysplasia. Hear. Rhythm..

[88] Santangeli P., Tung R., Xue Y., Chung F.-P., Lin Y.-J., Di Biase L., Zhan X., Lin C.-Y., Wei W., Mohanty S. (2019). Outcomes of Catheter Ablation in Arrhythmogenic Right Ventricular Cardiomyopathy Without Background Implantable Cardioverter Defibrillator Therapy. JACC Clin. Electrophysiol..

[89] Berruezo A., Acosta J., Fernández-Armenta J., Pedrote A., Barrera A., Arana-Rueda E., Bodegas A.I., Anguera I., Tercedor L., Penela D. (2016). Safety, long-term outcomes and predictors of recurrence after first-line combined endoepicardial ventricular tachycardia substrate ablation in arrhythmogenic cardiomyopathy. Impact of arrhythmic substrate distribution pattern. A prospective multicentre study. Europace.

